# Examining the influence of the domestic social robot anthropomorphism on users' perceived affinity via intelligent design process: robot social presence as a mediator

**DOI:** 10.3389/fpsyg.2026.1847715

**Published:** 2026-05-26

**Authors:** Tao Chen, Tianyu Cui, Yan Zhang, Zhuangzhuang Xu, Yunxiang Jia

**Affiliations:** 1 Department of Industrial Design, College of Publishing, University of Shanghai for Science and Technology, Shanghai, China; 2 Institute of Design Science, University of Shanghai for Science and Technology, Shanghai, China

**Keywords:** appearance anthropomorphism, interaction anthropomorphism, perceived affinity, robot social presence, the uncanny valley effect, intelligent design

## Abstract

Integrating social robots into daily life has elevated the demands for robot anthropomorphism to enhance Human-Robot Interaction (HRI). Through the intelligent design process, this study adapted the robot's social presence model to examine the mediating effect of robot social presence on the relationships between robot anthropomorphism and users' perceived affinity. The study adopted a 2 (appearance anthropomorphism: low *vs*. high) × 2 (interaction anthropomorphism: low *vs*. high) mixed factorial research method, with appearance anthropomorphism as the between-subjects factors, interface anthropomorphism as the within-subjects factor, social presence variables as the mediators, and users' perceived affinity as the dependent variable. Student participants (*N* = 218) were recruited for an online survey concerning this research topic. The results indicated the positive main effect of robot interaction anthropomorphism and interaction effect on users' perceived affinity, but not appearance anthropomorphism. The mediating role of perceived social presence was supported, and the uncanny valley effect was confirmed on interaction anthropomorphism. Robot stimuli were successfully manipulated via intelligent design processes. Robot interaction anthropomorphism positively influences users' perceived affinity, and the robot's social presence plays a crucial mediating role in the relationship. Significant interaction effect highlighted the potential application of domestic social robots with high appearance and high interaction anthropomorphism in future family scenarios.

## 1 Introduction

Recent decades have witnessed significant advancements in artificial intelligence (AI), sensor technology, and material science, which have propelled human-robot interaction (HRI) into an era (Licardo et al., 2024; Hentout et al., 2019). The evolution trend of intelligent robots has shifted from single-purpose industrial automation tools

to sophisticated service robots capable of performing complex tasks in business and domestic settings, such as food delivery or automated cleaning (Thylén et al., 2023; Zhou et al., 2024; Roesler et al., 2024). As service robots gradually enter people's public or private lives, a critical challenge emerges: Many users remain unfamiliar with their advanced technologies, diverse appearances, and interaction modalities. The lack of familiarity can hinder users' acceptance and intention to use, thereby limiting the benefits of these technologies in our daily lives (Roesler et al., 2024; Li et al., 2010). An early study indicated that the functionality of a robot is a key driver of intention to use service robots (Wirtz et al., 2018). Meanwhile, with the increasing interactions occur between humans and robots in modern lives, the social-emotional HRI aspects, such as the affinity of robot appearance and the digital user interface (UI), should also be considered critical design elements of robot services, developed in concert with its core functions to ensure a cohesive robot image (Wirtz et al., 2018; Heerink et al., 2010).

This paradigm transformation has foregrounded the concept of robot anthropomorphism, as our society embraces social robots in various application fields, such as healthcare, education, hospitality, domestic settings, and psychological research of HRI (Duffy, 2003; Roesler et al., 2021; Klebbe et al., 2022; Dubois-Sage et al., 2024). Further, anthropomorphism involves designing robots with human-like characteristics, such as physical appearance, facial expressions, emotions, or human behaviors to enhance robot affinity, facilitate more socially meaningful interactions (Duffy, 2003). To this end, robot scientists and engineers have developed a spectrum of anthropomorphic social robots to explore user attitudes and interaction efficacy. Examples such as machine-like robot Cozmo with emotional facial expressions (Law et al., 2021), service robot Pepper with human-like upper body for social interactions (Mishra et al., 2023), high Anthropomorphic robotic head Flobi for subtle facial expression research (Chen and Jia, 2023), and the lifelike robot Geminoid F with a realistic female body and face for social communications (Chesher and Andreallo, 2021). The demand for anthropomorphic social robots reflects a fundamental human desire: to develop robots to achieve high affinity and emotional resonance with users, foster more intuitive human-like interactions, and thereby improve overall acceptance and intention to use the social robots (Roesler et al., 2021; Tärning et al., 2025).

Prior research consistently demonstrated that high anthropomorphic robot designs in either appearance or interactions may enhance users' perceived affinity and positively influence intention to use public service robots. For instance, a highly anthropomorphic appearance with a human head and body of a grocery service robot positively enhances the intention to use (Roesler et al., 2024); the mimicry of human facial expressions by manipulating eye colors, pupil sizes, etc. may strongly evoke corresponding emotional responses in users (Tärning et al., 2025); and robots with specific design cues like anthropomorphic bodies and clothing designs enabled the robots to be perceived as humans with a specific gender (Dennler et al., 2025). Besides these insights, a significant gap persists in this field of research that few prior studies have systematically examined the influence of robot anthropomorphism on the perceived affinity and intention to use a domestic service robot. More importantly, the potential interaction effects of appearance and interaction anthropomorphism have rarely been examined simultaneously. Accordingly, this study contributed to filling the research gap by investigating the following research questions:

RQ1: Do robot appearance and interaction anthropomorphisms influence users' perceived affinity and intention to use a domestic service robot?
RQ2: Does the perceived social presence of an anthropomorphic robot mediate the relationship between robot anthropomorphism and users' perceived affinity?

The objective of this study is to examine the influence of the robot's appearance and interaction anthropomorphisms on users' perceived affinity and intention to use, as well as the mediating role of the robot's social presence in these relationships. This study also adopts an intelligent design process for early design reference and support, helping designers to conceive and make design decisions for anthropomorphic robot designs. The findings may offer significant insights for designing anthropomorphic robots that foster positive socio-emotional connections and enhance the intention to use domestic service robots.

## 2 Theoretical framework

**Figure 1:**
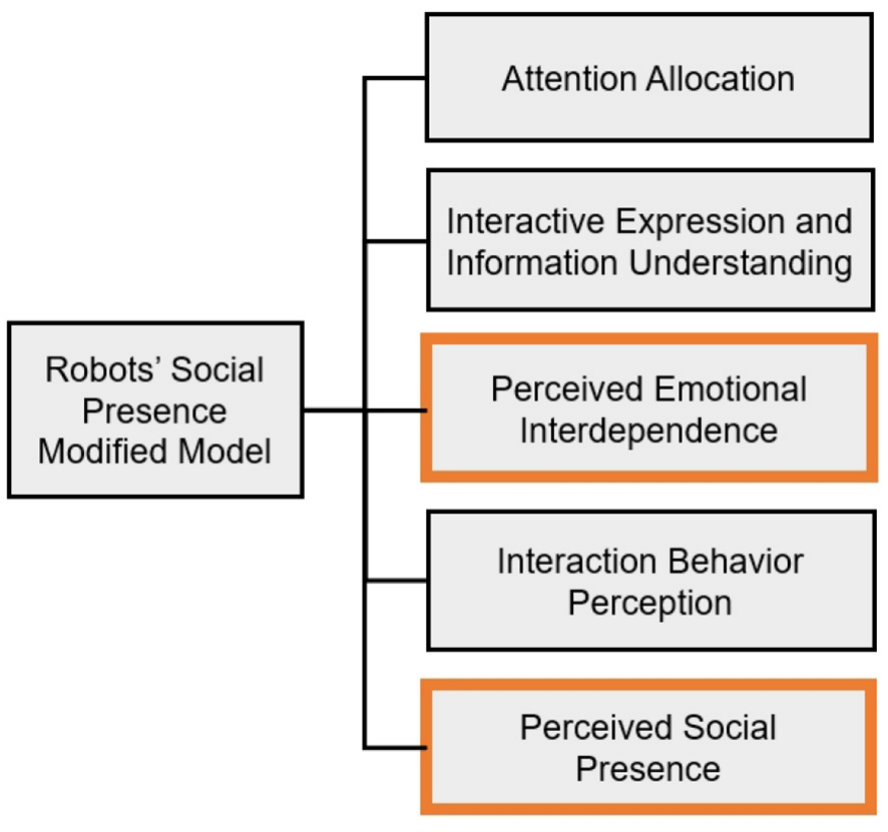
Robots' social presence modified model. Source: adapted from (Chen et al. 2023), p. 6.

The theoretical framework of this study is based on the Robots' Social Presence Modified Model (Chen et al., 2023), chosen for its emphasis on robots' social presence that highlights the social relationship and emotional connections in HRI. The general idea suggests that humans have social attributes in nature and have the inherent demand to maintain social relationships and belongings, which is typically fulfilled via behaviors such as staying in a social environment, interacting, and communicating with other people (Gunawardena, 1995). The Robots' Social Presence Modified Model (see Figure 1) has five factors representing robots' social presence from different dimensions. This study selectively incorporates the two most salient factors in the investigation of the research topic: perceived social presence (PSP) and perceived emotional interdependence (PEI) (marked in color in Figure 1). Social presence is defined as the extent to which one perceives the salience of others as real humans when being present and interacting with other people, and is one of the essential factors in maintaining social relationships (Short et al., 1976). Emotional interdependence refers to the extent to which humans and robots are mutually influential regarding emotions and attitudinal states (Salminen et al., 2018). These constructs are critical to this study, as robot anthropomorphic designs through human-like appearance and interaction behaviors are theorized to enhance users' perceived social presence and foster emotional interdependence (Chen et al., 2023; Jin and Youn, 2023).

From a theoretical perspective, PSP constitutes the primary perceptual level, triggered by initial sensory cues, including physical appearance, voices, and gestures. It emphasizes the human user's subjective awareness of the robot as a distinct social entity within a shared environment (Short et al., 1976; Monfardini et al., 2016). In contrast, PEI represents a secondary relational level that emerges after social presence has been established. It focuses on the affective mutual influence between agents, encompassing the synchronization of emotions, attitudes, and moods within HRI (Huang, 2003; Chen et al., 2023). Furthermore, Chen et al. (2023)'s study of the measurement development of robot social presence indicated that PSP and PEI could be formed via initial visual perceptions of social robots such as appearance and facial expressions, shape human's first impressions toward social robots, while other factors may require deep interactions to perceive. PSP and PEI are conceptually distinct yet equally critical factors in shaping the initial quality and outcomes of HRI, and thus, are examined in this study.

In addition to the Robots' Social Presence Modified Model, this study draws upon the Theory of Planned Behavior (TPB) as a foundational framework for understanding behavioral intentions and constructing robot anthropomorphism-related factors. The TPB provides a robust theoretical basis for predicting the relationship between users' attitudes and intention to perform a behavior (Ajzen, 1991). Within our research context, users' attitude of perceived affinity (PA) toward an anthropomorphic robot may positively influence users' behavior of intention to use the social robots.

**Figure 2:**
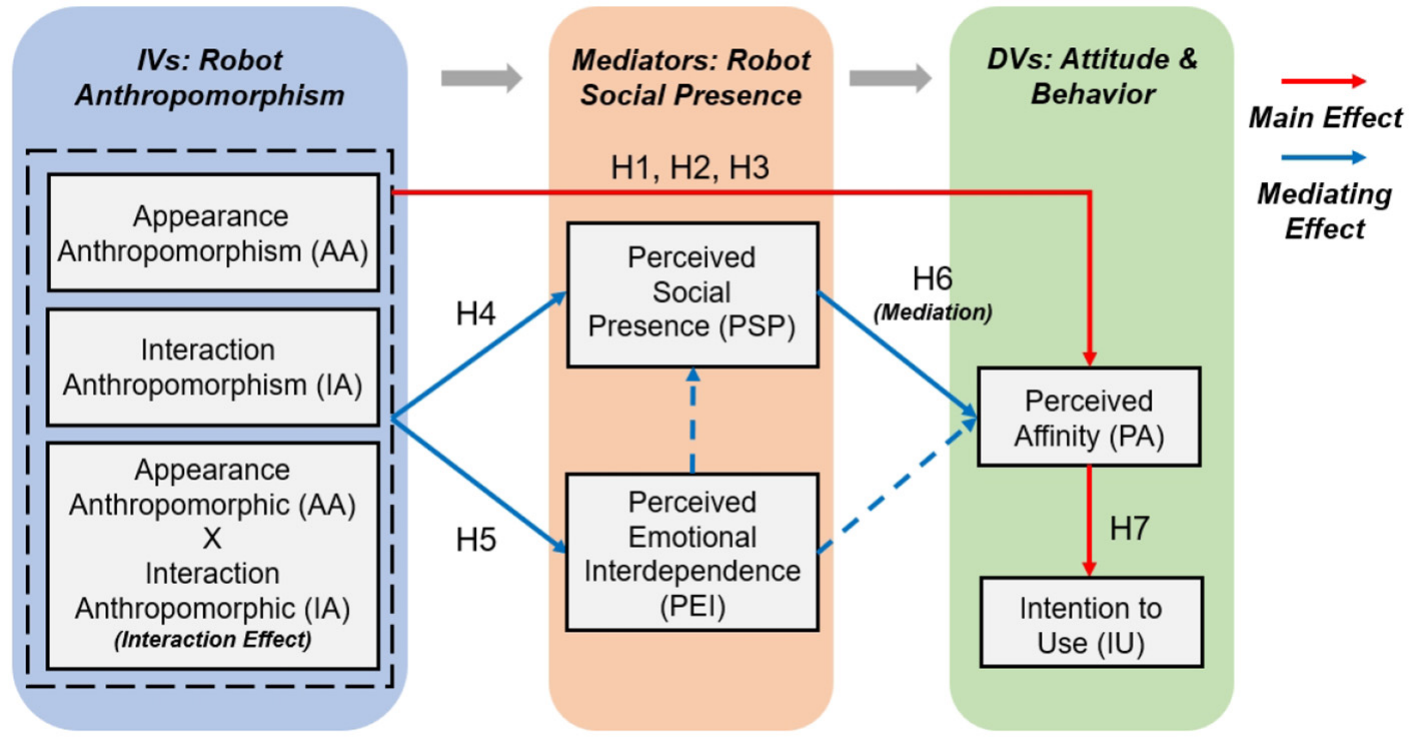
Proposed initial theoretical framework of robot anthropomorphism influence users' attitudes via robot social presence.

Hence, this study posits a mediational model in which the robot's perceived social presence and perceived emotional interdependence play mediating roles in influencing the relationships between robot anthropomorphism and perceived affinity and further influencing intention to use. The theoretical framework of this study is shown in Figure 2. A comprehensive literature review is presented in the following sections to provide the theoretical and empirical justification for each variable.

## 3 Literature review

### 3.1 Robot anthropomorphism

Anthropomorphism is defined as the practice of imputing human characteristics, such as appearance, cognitive or emotional features, to non-human entities to rationalize their behaviors (Duffy, 2003). Robot anthropomorphism, thus, refers to the intentional design of robots with physical humanoid features, emotional intelligence, and interactive capabilities (Duffy, 2003; Roesler et al., 2021). This design philosophy has gained prominence with the broader applications of robots from industrial contexts to social scenarios with increased needs for services and companionship (Matheson et al., 2019; Fong et al., 2003). The viability of this transition was demonstrated in prior study where an anthropomorphic industrial robot was placed in a public exhibition environment to interact with humans, successfully engaged in human social scenarios to communicate with, provide services, and received positive feedback from users, signaled the broader potential for anthropomorphic robots in social support roles (Capy et al., 2022b).

The trend of robot anthropomorphism could be explained from two primary lenses: functional and social. Regarding the functional aspect, anthropomorphic structures are designed for the physical benefits of humans, such as artificial limbs in medical rehabilitation to provide physical support and movements (Fong et al., 2003; Xu and Todorov, 2016; Garcia-Gonzalez et al., 2022). Conversely, regarding the social aspect, robot anthropomorphism, specifically appearance anthropomorphism (AA) and interaction anthropomorphism (IA), fosters familiarity via simulating human appearance and intuitive interaction patterns, thereby enhancing human-robot interaction (HRI) (David et al., 2022; Dang and Liu, 2024). This study is situated exclusively within this social aspect in robot anthropomorphism of AA and IA, and elaborated in subsequent sections.

### 3.2 The uncanny valley effect

**Figure 3:**
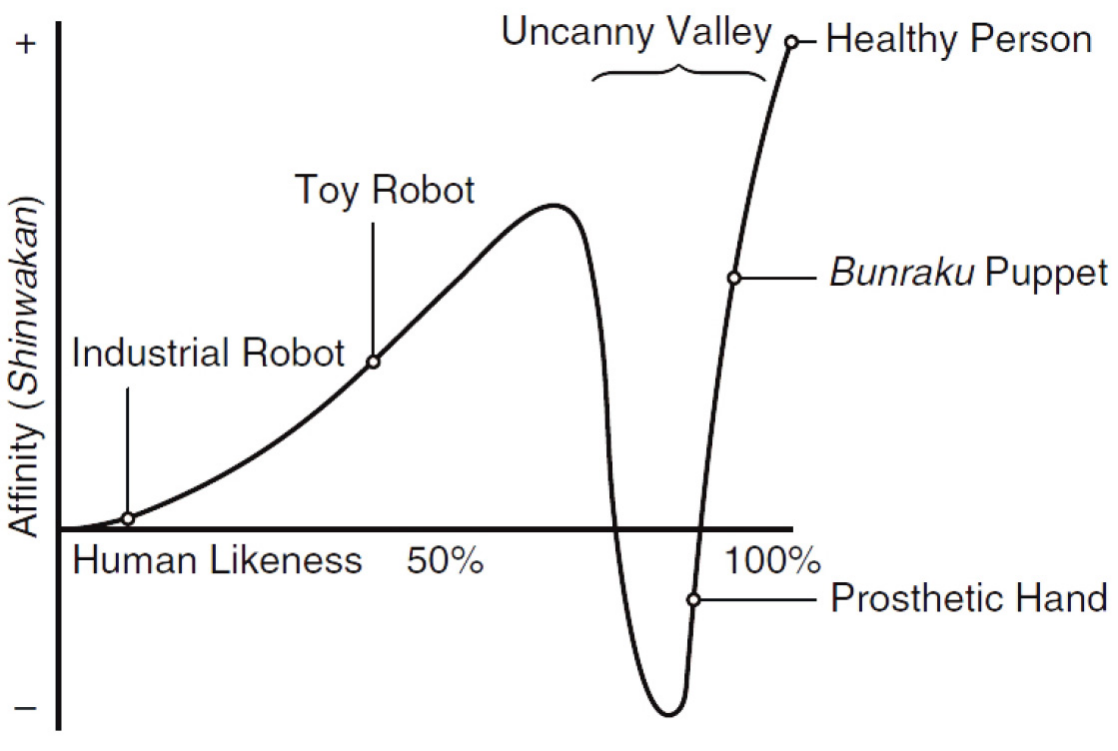
Mori's the uncanny valley. Source: (Mori et al. 2012), p. 99.

The uncanny valley effect, first posited by Mori (1970) and Mori et al. (2012), is a critical consideration in studying robot anthropomorphism. The theory assumes a non-linear relationship between robot anthropomorphism and users' PA, that users' PA for robots generally improve with the enhancement of the human-likeness of the robots. However, the preference curve would experience a drastic decline into a “valley” when the robot is at almost but not fully human-like levels (see Figure 3). Such close-to-realistic levels evoke feelings of eeriness or strangeness, leading to negative users' PA for robots (Duffy, 2003; Kätsyri et al., 2015).

Evidence from empirical research has confirmed the uncanny valley effect on people's PA for anthropomorphic robots. However, the specific border for the uncanny valley is still unclear and may vary depending on different conditions (Kätsyri et al., 2015; Kim et al., 2022; Mathur et al., 2020). For example, Kätsyri et al. (2015) study suggested that mismatching stimuli or atypical features may lead to the uncanny valley effect, such as real human eyes on a fake face, or a human-like face with disproportionate eyes. Similarly, Woods (2006) study concerning children's perceptions of robots also indicated that children perceived human-machine robots as more intimate than human-like robots, indicating an uncanny valley effect that children felt discomfort when interacting with highly human-like robots, but still different from humans.

Given this body of evidence, this study will also evaluate the uncanny valley effect that potentially negatively influences users' PA for higher levels of anthropomorphic robots manipulated in this study.

### 3.3 The perceived affinity for robots

Perceived affinity serves as the primary dependent variable in this study. According to Franke et al. (2019), PA for anthropomorphic robots could be defined as the tendency for users to actively cater to the interactions with robot technologies, including functionalities and social interactions. In the context of social robots, PA is an indicator of how users can understand and interact with robots in an efficient and effective way, and it is also the users' positive perception of and emotional attachment to the robots (Wessel et al., 2020; Belanche et al., 2021). With the advancement of social robots, besides providing functionalities, social and emotional needs, such as warmth and affinity, have also been assumed to improve the acceptance of and intention to use social robots (Wirtz et al., 2018). Thus, the significant role of affinity for robots has been studied and highlighted in recent studies (e.g., Roesler et al., 2024; Chang, 2024; Roesler et al., 2023; Sejima et al., 2021) to connect social robot attributes to acceptance and intention to use (IU).

However, user attitudes toward technology are often influenced by demographic factors. A previous study indicated that gender difference has a significant connection with users' experience with new technologies (Müller-Abdelrazeq et al., 2019). Compared to females, males may demonstrate higher interests in new technologies, leading to their more positive attitudes (Claßen, 2013; Broos, 2005). Thus, gender effects may also exist in users' attitudes and perceptions of robots' affinity, and may potentially interfere with the results of the study (Nomura et al., 2004).

### 3.4 Appearance anthropomorphism influences users' perceived affinity

The visual perception of a robot's appearance constitutes one of the most fundamental factors of anthropomorphism (Hegel, 2012). People's initial reactions to anthropomorphic robots are often instantaneous, shaped by hints and key features of the visual cues of a robot's appearance, such as human-like head, limbs, and body (Goetz et al., 2003; Aarts and Dijksterhuis, 2000). The first impression is vital, as it establishes a cognitive mental model that informs users' expectations of the robot's capabilities and functionalities, even before HRI begins (Powers and Kiesler, 2006; Kiesler and Goetz, 2002). Consequently, a robot's appearance is a powerful initial signal that affects people's perceptions of the robot's functionalities and may further affect users' PA and IU (Hegel, 2012; Goetz et al., 2003).

Empirical research consistently demonstrates that a robot's appearance significantly influences users' PA. Design methods such as avoiding angular shapes and using white color systems to form a soft impression may enhance the PA of the robots (Shao et al., 2019). Goetz et al. (2003) argued that a robot's appearance and behavior that match its occupations or task scenarios may improve users' preference and affinity and enhance the collaborations in HRI. This principle of task-congruence is confirmed by prior empirical studies across different domains. For example, Złotowski et al. (2020) study on the relationship between robot anthropomorphism and application domain indicated that an appearance anthropomorphic robot, compared to a machine-like robot, fosters familiarity and is preferred for occupations focused on social service tasks such as consulting and caregiving. Roesler et al.'s (Roesler et al., 2024) study revealed that people prefer social robots with high anthropomorphism when using a service robot for grocery shopping, supporting the importance of task-congruent appearance anthropomorphic design in enhancing users' PA and IU of social robots. A cross-culture study of robot appearance from Li et al. (2010) also indicated that, compared to a machine-like robot, an appearance anthropomorphic robot is more preferred, as humanoid attributes enhance people's familiarity to the robot, potentially match its tasks as security guard or tour guide, and lead to a higher PA.

Thus, regarding domestic social robots investigated in this study, an AA social robot is preferred according to the domestic social scenario, and may positively evoke users' PA. However, due to the uncanny valley effect, a low AA social robot is more preferred than a high AA social robot in terms of PA. According to the preceding literature review, hypothesis 1 is proposed:

H1: AA of a domestic social robot will positively influence users' PA. Specifically, a domestic social robot with a low AA perceives higher affinity than one with a high AA.

### 3.5 Interaction anthropomorphism influences users' perceived affinity

IA in this study refers to the robot anthropomorphism of facial expression interaction. Interactions among humans include non-verbal communication via facial expressions, conveying abundant information and emotions (Tärning et al., 2025). As robots become closer to interpersonal roles and are integrated into more social scenarios, the demand for their abilities to emulate human-like emotional expression is growing (Tärning et al., 2025; Torre et al., 2020). According to the Theory of Mind (ToM) and related neuroimaging studies, Dubois-Sage et al. posited that, similar to the activation of ToM among human-human interactions, IA could also be enabled during HRI and effectively elicit human mental states and affective responses, such as emotions (Premack and Woodruff, 1978; Dubois-Sage et al., 2023). Robotic face design plays a vital role in shaping effective HRI, as it goes beyond technical function, shaping perceptions of identity, emotion, and even moral integrity (Chesher and Andreallo, 2021). Consequently, a well-designed robotic facial expression interaction system can attract users' attention, promote natural and comfortable communication, foster positive attitudes, and enhance users' PA and IU (Lehmann et al., 2016; Zebrowitz and Montepare, 2008).

Empirical evidence consistently demonstrates that specific design choices in robotic facial expressions significantly influence users' PA and emotional response. Wairagkar et al. (2022) found that a social robot with a hybrid face, which combines a physical faceplate with a digital display of simple expressions, elicited positive face-sensitive neurophysiological potentials in electroencephalography, and could deliver positive emotions and improve HRI, elevate users' satisfaction and users' PA. Lehmann et al. (2016) examined users' preference for robot interaction anthropomorphism by designing several facial expressions with different emotions for a humanoid service robot called R1. The results indicated that users preferred iconic facial expressions with human-featured eyes and eyebrows to abstract ones, and the childish features of iconic faces may remind users of lovely cartoon characters, and improve their PA. Indeed, a study manipulating face attributes of several real world social robots confirmed that significantly increased perceptions of cuteness and trustworthiness, which in turn fostered greater emotional interaction and higher PA (Chen and Jia, 2023).

Thus, regarding domestic social robots investigated in this study, an IA social robot is generally preferred to facilitate intuitive social interactions and positively evoke users' PA. However, due to the uncanny valley effect, a low IA social robot is more preferred than a high IA one in terms of users' PA. According to the preceding literature review, hypothesis 2 is proposed:

H2: IA of a domestic social robot will positively influence users' PA. Specifically, a domestic social robot with a low IA perceives higher affinity than one with a high IA.

Besides the main effects of AA and IA, the interaction effects of AA and IA are also expected to influence users' PA. A previous study Jia and Chen (2022) pointed out that abstract (*vs*. concrete) facial expressions on a robot with a head (*vs*. without a head) are preferable, indicating that the interaction effect of AA and IA existed. AA and IA may work together to influence users' attitudes of affinity. Again, due to the uncanny valley effect, specifically, a domestic social robot with high AA and high IA may be perceived as the lowest affinity by users, from low to high, followed by low AA and high IA, high AA and low IA, and then low AA and low IA. According to the preceding literature review, hypothesis 3 is proposed:

H3: The interaction effects of AA and IA of a domestic social robot will positively influence users' PA. Specifically, a domestic social robot with high AA and high IA may perceive the lowest affinity from users, from low to high, followed by low AA and high IA, high AA and low IA, and then low AA and low IA.

### 3.6 The mediating role of robot social presence

Social presence is defined as the subjective feeling of being accompanied by a “real” person, characterized by perceived access to their thoughts and emotions (Biocca, 1997). PSP is a crucial psychologically influential factor in HRI. It has previously been explored in specific no-human-present scenarios to enhance someone's perceptions of others' presence, such as in a human-AI interaction scenario to improve PSP of an virtual AI chatbot for higher use intention (Jin and Youn, 2023), and in an online group learning scenario to elevate SP via virtual reality (VR) technics to enhance students' learning motivations (Kreijns et al., 2022). In line with the ToM regarding HRI, social presence is particularly relevant for explaining the effects of robot anthropomorphism (Premack and Woodruff, 1978; Dubois-Sage et al., 2023). Empirical evidence confirmed that high anthropomorphic robots with real human attributes, compared to low ones, could enhance users' PSP and received more positive attitudes toward the social robots (Kiesler et al., 2008). Further, social robots which could perform human-like behaviors, such as face-to-face interactions, are more likely to form effective PSP among users (Oh et al., 2018). For example, an empirical study from Liu et al. (2025) argued that, by observing images and videos regarding anthropomorphic robots' appearance and limb gestures, higher PSP was perceived by users for highly anthropomorphic robots with human-like appearance and fluent movements in accommodation service scenes. Premathilake and Li (2024) study regarding customers' evaluations of hotel social robots indicated that higher physical and voice interaction anthropomorphic social robots positively influence customers' PSP, leading to their IU.

A second key psychologically influential factor investigated is perceived emotional interdependence (PEI), defined as the extent to which the emotions and attitudes of a person and a social robot influence each other (Salminen et al., 2018). Emotional interaction is fundamental to HRI. It forms PEI and increases users' willingness to interact with anthropomorphic robots (Chen et al., 2023). Prior study from Li and Zhao (2023) supported that a humanoid robot with designed robotic arms could express positive emotions to influence users' willingness to interact with the robot, enabling users to feel they are interacting with a real person, and further enhance their PA. Similarly, Urakami (2023)study supported the idea that the cool appearance study also suggested that a robot's facial expressions could evoke emotional resonance with users; for instance, a robot with neutral emotions is preferable, while a sad expression can prompt users' empathetic, helpful responses. These findings collectively suggest that when robots display AA and IA, they are merely seen as tools but as interactive partners capable of forming emotional connections. According to the preceding literature review, hypotheses 4 and 5 are proposed:

H4: AA (a), IA (b), and the interaction effect of AA and IA (c) of a domestic social robot will positively influence users' PSP.
H5: AA (a), IA (b), and the interaction effect of AA and IA (c) of a domestic social robot will positively influence users' PEI.

A higher level of PEI could be fostered via increased mutual understanding and emotional interactions, further enhancing users' PSP (Chen et al., 2023; Huang, 2003). Hence, PSP mediates the relationship between PEI and PA. Thus, hypothesis 6a is proposed:

H6a: Users' PSP will mediate the relationship between users' PEI and users' PA for a domestic social robot.

As the increasing demands for robots enter the social scenarios for communications and services, the ToM is activated that human users perceive anthropomorphic robots with certain human attributes to consider them as social partners (Damiano et al., 2012; Dubois-Sage et al., 2023). Robot anthropomorphism characteristics, such as human-like heads, faces, limbs, facial expressions, and gestures, play essential roles in evoking emotional connections, facilitating users' perception process of SP during HRI, providing companionship to alleviate users' loneliness, and enhancing users' PA (Walters et al., 2008; Ke et al., 2020; Capy et al., 2022a). Thus, PSP plays a mediating role in influencing the relationship between robot anthropomorphism and users' PA. According to the preceding literature review, hypotheses 6b to 6d are proposed:

H6b: Users' PSP will mediate the relationship between AA and users' PA for a domestic social robot.
H6c: Users' PSP will mediate the relationship between IA and users' PA for a domestic social robot.
H6d: Users' PSP will mediate the relationship between the interaction effect of AA × IA and users' PA for a domestic social robot.

### 3.7 Users' perceived affinity influences users' intention to use

Drawing on Ajzen's TPB, which posits that users' attitudes positively influence intention to behave (Chen et al., 2023). In the context of domestic service robots, previous studies indicated that robot anthropomorphism may improve users' attitude toward PA, and further positively influence users' IU of the robot (Ivanov and Webster, 2019). For example, Huang et al. (2024) study supported the idea that the cool appearance and interaction courtesy of hospitality service robots will foster positive affect attitudes such as users' PA, and further influence users' IU of the service robots. Similarly, Cha (2020) study also suggested that during the COVID-19 pandemic period, people in Korea held positive attitudes toward restaurant service robots regarding utility and appearance attractiveness, which improved users' perceived enjoyment and affinity, and further led to their intention to interact and use the service robots. According to the preceding literature review, hypothesis 7 is proposed:

H7: Users' PA will positively influence users' IU of a domestic social robot.

### 3.8 Intelligent design process for robot anthropomorphic designs

Intelligent design leverages state-of-the-art technologies such as VR, AI, big data analytics, and human-computer interaction (HCI) to achieve its objectives throughout the entire product design process (Zhang et al., 2017; Zhang, 2022). Recent research has demonstrated the capability of artificial intelligence-generated content (AIGC) image tools in inspiring robot appearance design, notably enhancing efficiency and novelty via the intelligent design process. For instance, Ren et al. (2024) developed and trained an AI model specifically for the design of public health social robots. Their findings validated the integration of AI tools in the intelligent design process to generate high-quality reference images for the aesthetics of robot appearance, guided by the principles of Kansei engineering. Furthermore, by adopting the Theory of Inventive Problem Solving (TRIZ), Yang et al. (2025) study established an intelligent design method for designing aging service robots using AIGC image tools. The finding also indicated the appropriateness of applying this intelligent design method in enhancing design quality and innovation for aging service robot design. Consequently, incorporating AIGC image tools in the intelligent design process could potentially enhance design efficiency and novelty of robot anthropomorphic designs, and will be implemented in the manipulation process of robot stimuli in this study.

## 4 Method

### 4.1 Research design

The research was proposed and conducted in a multimedia intelligent laboratory at a university in East China. The hypotheses were tested via an experimental study conducted online using the Wenjuanxing survey platform (https://www.wjx.cn/). The study employed a 2 (AA: low *vs*. high) × 2 (IA: low *vs*. high) mixed-factorial experimental design with AA as the between-subjects factor, IA as the within-subjects factor, social presence variables (PSP and PEI) as the mediators, and users' PA and IU as the dependent variables.

### 4.2 Stimulus development: an intelligent design process

The domestic service robot was selected as the topic of the stimulus, as service robots are popular and familiar to the majority of the population in China, and have already been utilized in business environments for consulting, food and courier delivery. Intelligent design processes using an AIGC image tool and a facial expression APP were used in this stimulus development to improve design quality and efficiency.

**Figure 4:**
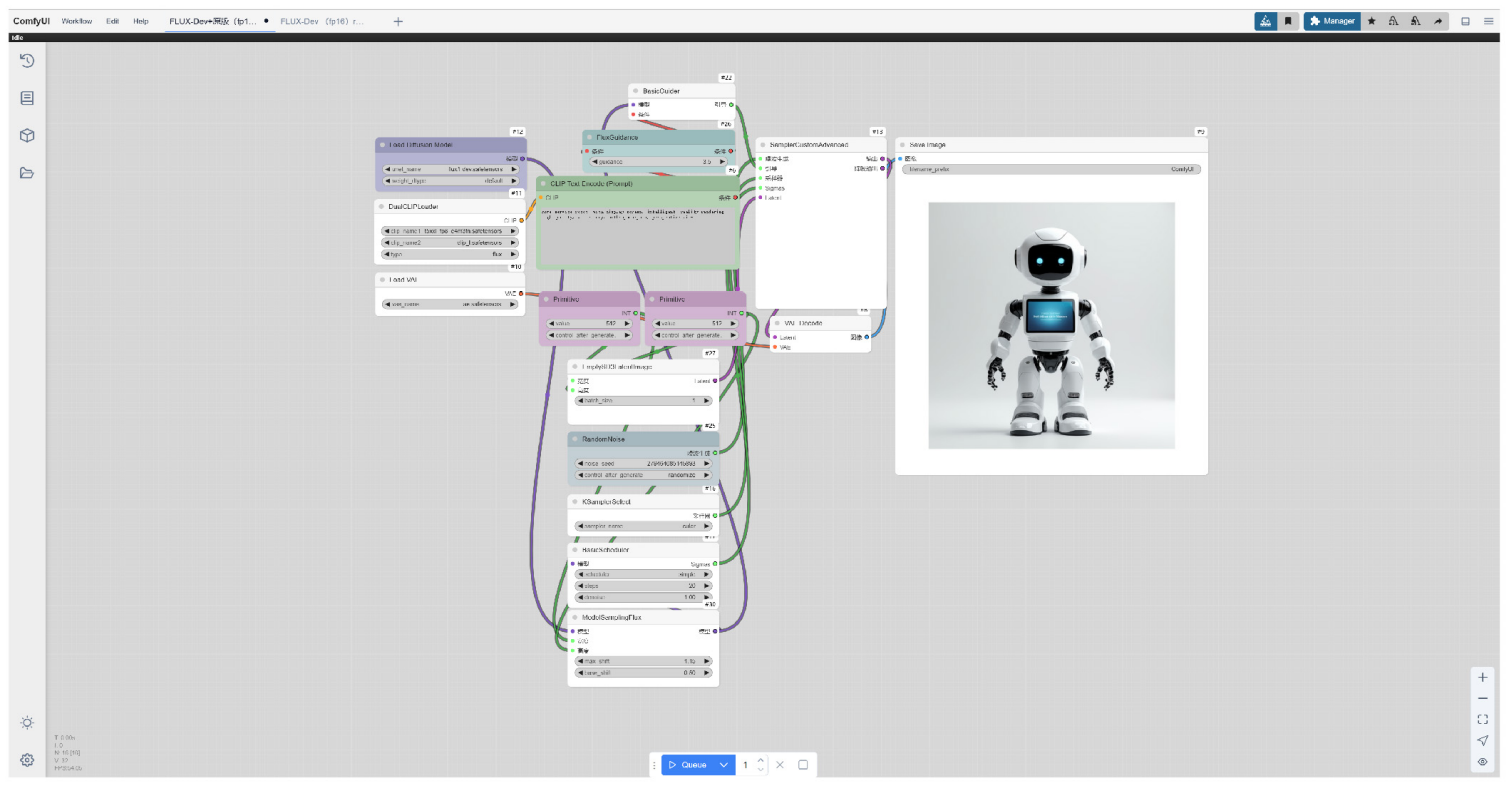
Stable diffusion with comfy UI demonstrating the prompts and parameter settings.

For the stimulus development of two levels of robot AA, a human-AI collaboration intelligent design method was used, as AI was used to generate high-quality robot images, and a human researcher was invited to revise the image details to meet the design criteria. In the first step, the AIGC image tool Stable Diffusion with Comfy UI was used in this study to generate preliminary robot images with high AA as references. The AI models, Flux1-dev, SD 3.5, and Deliberate v5 were used. The prompts are “home service robot, with display screen, intelligent, reality rendering, high quality, white body, anthropomorphic, perspective view” with minor changes; all other settings were default. The prompts and all the details of the parameters and settings are shown in Figure 4. A desktop with an NVIDIA 4090 graphic card was used to run the AIGC image tool. More than 100 images with 512 × 512 pixels were generated by Stable Diffusion within an hour. Due to the nature of AIGC image tools that randomly generate images, not all generated images can meet the stimulus manipulation criteria. Thus, a panel of three design-majored experts was invited to help screen and select the AI-generated images that best match the design criteria. The ideal image for a high AA robot is a white, complete upper body robot with a digital screen on the face, standing on a moving chassis with a 45-degree perspective view and a gray background. Finally, three AI-generated robot images were selected for further manipulation.

In the second step, one of the researchers in this study used the graphic design software, Adobe Photoshop, to manipulate the selected robot images into two AA levels (high and low). Regarding the high-level anthropomorphism, the robot images were mainly adopted from AI-generated images with minor changes, such as changing the background to gray and cleaning unexpected and strange content. Regarding the low-level anthropomorphism, the robot images were adopted from the high-level ones, replacing the upper body with a rounded-edge box and a digital screen as the face. Finally, a set of two robot images with low and high anthropomorphism levels was selected, used Stable Diffusion with Comfy UI, and the SUPIR model to enlarge the initial 512 × 512 pixels into 2,048 × 2,048 pixels images with no quality loss for the manipulation check.

For the stimulus development of two levels of robot IA, regarding high-level anthropomorphism, the intelligent design method adopting the Memoji App from iPhone iOS was used to generate five detailed facial expressions: smile, greeting, confused, upset, and sleeping. By scanning real human faces, the Memoji App could generate respective meticulous animated facial expressions instantly. The facial expressions were snapshot and dealt with Adobe Photoshop to clean the background and preserve the Memoji face expressions only. Female heads with neutral brown long hair and yellow skin were selected in this study, as previous studies indicated the appropriateness of female attributes (e.g., friendlier, higher emotion expressions) in robots for social service scenarios (Borau et al., 2021; Ding et al., 2025). Regarding low-level anthropomorphism, one of the researchers in this study used the graphic design software, Adobe Illustrator, to manipulate low-level interface anthropomorphism. The design criteria were to use basic shapes, such as lines and circles, to depict eyes and mouths, and five simple facial expressions were designed: smile, greeting, confused, upset, and sleeping.

**Figure 5:**
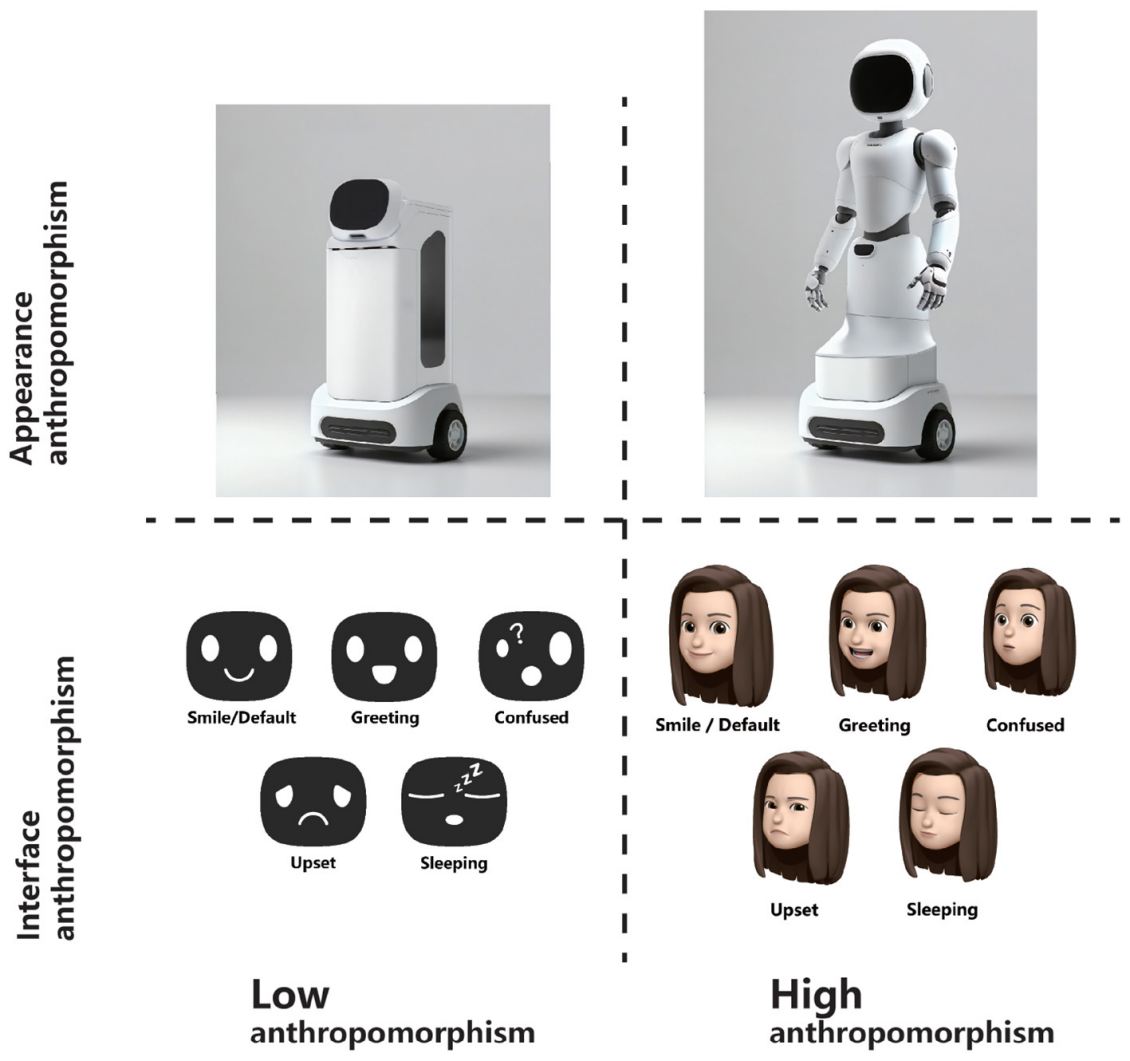
Manipulated AA and IA stimuli images.

The intelligent design processes using new technologies demonstrated high quality and efficiency for robot image manipulations. All the manipulated images were finished within two days and are shown in Figure 5.

### 4.3 Pretest-manipulation check

**Figure 6:**
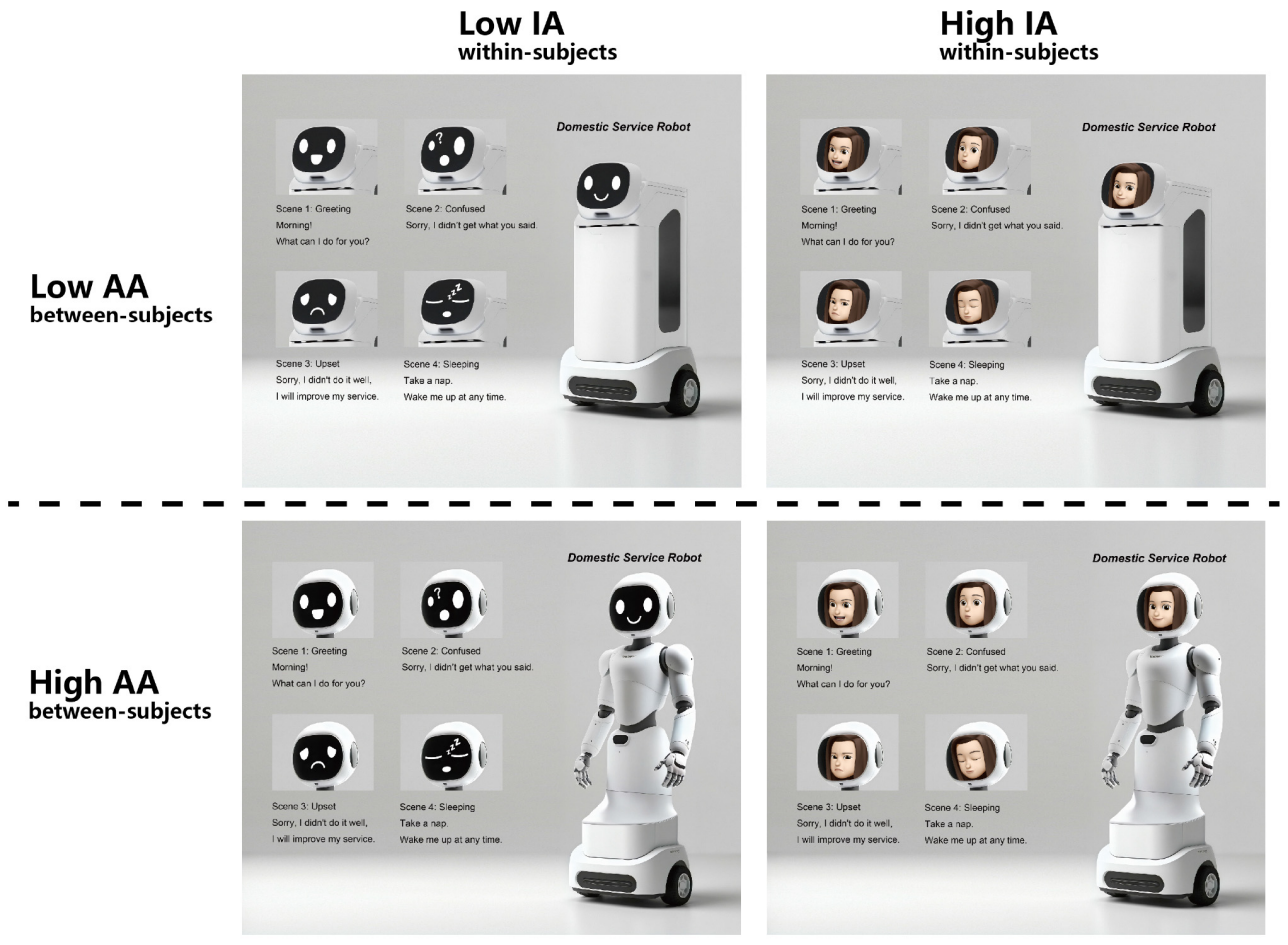
Overview of the final stimuli images.

The pretest aimed to validate the manipulated anthropomorphic levels of AA (Low *vs*. High) and IA (Low *vs*. High) of the two pairs of stimuli. Two pairs of stimuli were validated with 55 undergraduate students at a university in East China. The appearance anthropomorphism scale (3 items, 7-point Likert scale) was adapted from David et al. (2022) social robot anthropomorphism scale (SRA) and rephrased into AA and IA conditions (see Appendix A). Participants responded to anthropomorphic scale measurements of robot AA and IA across two levels following the stimulus image. For the robot anthropomorphism manipulations to be successful, the means of both the low level of AA and IA of the stimuli must be significantly smaller than that of the corresponding high level of AA and IA of the stimuli (*M*_*AALow*_<*M*_*AAHigh*_; *M*_*IALow*_<*M*_*IAHigh*_). A series of paired samples t tests were conducted. Results indicated that the manipulations were successful, all the stimuli met the required criteria as expected: AA manipulations: *M*_*AALow*_ = 3.20 < *M*_*AAHigh*_ = 5.82, t = –13.733, *p* < 0.001; and IA manipulations: *M*_*IALow*_ = 4.70 < *M*_*IAHigh*_ = 5.84, t = –6.806, *p* < 0.001. Thus, the domestic service robot anthropomorphism manipulations of both appearance and interface were deemed successful. As a result of the pretest, a set of four domestic service robot images was finally combined using Adobe Photoshop, showing as follows: Low AA × Low IA, Low AA × High IA, High AA × Low IA, and High AA × High IA. See Figure 6 for the overview of stimuli.

### 4.4 Sample

The hypothesized relationships were tested via an online survey. The survey was constructed and posted on the Wenjuanxing survey platform. The questions in the survey were originally composed in English and then translated into Chinese, and proofread by an English-majored Chinese expert to ensure the accuracy of translations. Student participants were recruited from a university in East China. Young generations, characterized as digital natives, are growing with the continuous exposure to diverse frontier technologies. Thus, young generations typically possess higher technology literacy, and a greater tendency to adopt new technologies and learn related knowledge such as AI and social robots (Vitezić and Perić, 2021; Fu et al., 2024). They generally holds a more positive attitude toward AI and social robots, and believed the new technologies would make the future better (Méndez-Suárez et al., 2023). The selection of a student sample ensured a baseline level of technological familiarity. A survey QR code was sent to the corresponding class WeChat groups or posted during the courses by the professors to invite the student participants to complete the online survey. Upon completion, participants received 2 CNY as incentives.

A total of 249 student participants joined in the survey research. Questionnaires with an answering duration of less than one minute, more than 10 minutes, or a consistent selection of the same number for all items in a question were screened out. Finally, 218 valid questionnaires were collected for further data analysis. As AA is the between-subject variable, of the 218 participants, 103 were randomly assigned to low AA, and 115 to high AA conditions. The usable samples consisted of 121 females (55.5%) and 97 males (44.5%), aged from 18 to 25 years old (M = 21.47, SD = 1.303). Regarding academic standing, 91.3% of the respondents were undergraduate students, and 8.7% were graduate students. Academic majors within Product Design (34.9%) represented the majority, followed by Computer Science (18.8%), Industrial Design (14.2%), Robot Engineering (14.2%), Animation (6.9%), English (4.6%), and others (6.4%). See Table 1 for the sample descriptions.

**Table 1:** Sample descriptions for pretest and main test.

	Sample descriptions	
*Pretest*	Participant	55 students at a university in East China
	Gender	Females: 41 (74.5%)
		Males: 14 (25.5%)
	Age	20–29, Mean = 22.78, SD = 1.969
	Grade	Junior: 18 (32.7%)
		Senior: 5 (9.1%)
		First year graduate student: 24 (43.6%)
		Second year graduate student: 5 (9.1%)
		Third year graduate student: 3 (5.5%)
*Main test*	Participant	218 students at a university in East China
	Gender	Females: 121 (55.5%)
		Males: 97 (44.5%)
	Age	18–25, Mean = 21.47, SD = 1.303
	Academic standing	Undergraduate student: 24 (91.3%)
		Graduate student: 24 (8.7%)
	Academic major	Product design (34.9%)
		Computer science (18.8%)
		Industrial design (14.2%)
		Robot engineering (14.2%)
		Animation (6.9%)
		English (4.6%)
		Others (6.4%).

### 4.5 Instrument and data analysis

The survey includes four variables, 20 scale items measuring users' PA, PSP, PEI, and IU of the service robots. The measures for all variables were chosen from previously developed scales with acceptable reliabilities. All the reliability values of the scale measurements were reported from the respective sources and shown in Appendix A.

A repeated measures ANCOVA was used to analyze the quantitative survey data and determine the influences of 2 (AA: low *vs*. high) × 2 (IA: low *vs*. high) mixed factors on users' PSP, PEI, and PA for the service robots. Then, the bootstrapping PROCESS macro for SPSS was used to predict the mediating effect of PSP on the relationship between PEI and users' PA. Single linear regression analyses were used to predict the effect of users' PA on IU of the service robot. IBM SPSS 27 software and its plugin application PROCESS macro for SPSS were employed for data analysis and result reporting.

## 5 Results

### 5.1 Reliability and validity

The internal consistency of all the scales was assessed using Cronbach's alpha. The analyses confirmed high reliability for each variable, as all alpha coefficients exceeded the recommended 0.70 threshold: PA, 0.893–0.956; PSP, 0.844–0.878; PEI, 0.828–0.896; IU, 0.877–0.935.

To examine construct validity, factor analyses with the varimax rotation method were conducted. The results supported the unidimensionality of the measures, with Kaiser-Meyer-Olkin (KMO) values exceeding 0.60, except that the robot social presence variables, PSP and PEI, were generally in the same loading. This issue will be addressed in the respective test of hypothesis.

### 5.2 Test of hypotheses

The hypotheses regarding main effects were tested using repeated measures ANCOVA. The model included AA as the between-subjects factor, IA as the within-subjects factor, PA as the dependent variable, and gender as the covariate.

**Figure 7:**
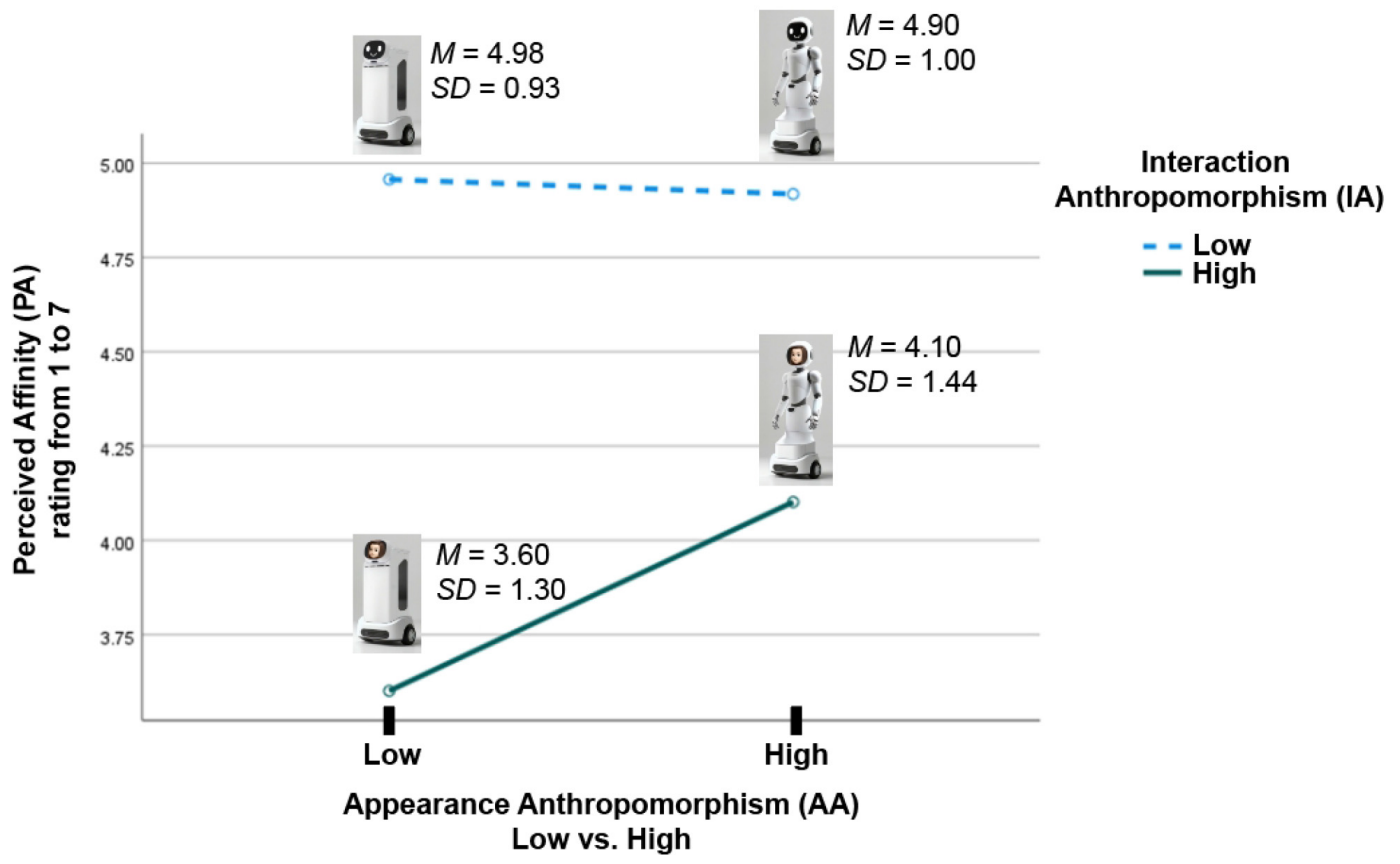
Interaction effect of AA and IA on PA.

The result revealed a nonsignificant main effect regarding the between-subject effects of AA on PA [*F*_(1/215)_ = 2.899, *p* = 0.09, η^2^ = 0.013]; thus, H1 was not supported. The result revealed a significant main effect regarding the within-subject effects of IA on PA [*F*_(1/215)_ = 4.348, *p* = 0.038 < 0.05, η^2^ = 0.02], Specifically, users had a more positive PA of low as opposed to high IA [*M*_*LowIA*_ = 4.94, *SD* = 0.96, *M*_*HighIA*_ = 3.87, *SD* = 1.40], supporting H2. H3 proposed the positive interaction effect of AA and IA on users' PA. Result from the repeated measures ANCOVA revealed a significant interaction effect [*F*_(1/215)_ = 8.546, *p* = 0.004 < 0.05, η^2^ = 0.038]. Specifically, users' PAs were higher in low IA conditions than in high IA conditions. While in high IA conditions, users' PA was higher in the high AA condition than in the low AA condition. [*M*_*LowAA*×*LowIA*_ = 4.98, *SD* = 0.93, *M*_*LowAA*×*HighIA*_ = 3.60, *SD* = 1.30, *M*_*HighAA*×*LowIA*_ = 4.90, *SD* = 1.00, *M*_*HighAA*×*HighIA*_ = 4.10, *SD* = 1.44]. The scores of perceived affinities from low to high are (1) Low AA × High IA = 3.60, (2) High AA × High IA = 4.10, (3) High AA × Low IA = 4.90, and (4) Low AA × Low IA = 4.98. The plot demonstrating the significant interaction effect of AA and IA is shown in Figure 7. The details are not fully consistent with H3; thus, H3 was partially supported.

The hypothesized influence of robot anthropomorphism variables on PSP and PEI was tested through repeated measures ANCOVAs, including AA as the between-subjects factor, IA as the within-subjects factor, PSP and PEI as the dependent variables, and gender as the covariate.

The result demonstrated a nonsignificant effect regarding the between-subject effect of AA on PSP [*F*_(1/215)_ = 0.082, *p* = 0.774, η^2^ = 0.00]; thus, H4a was not supported. The result demonstrated a significant effect regarding the within-subject effect of IA on PSP [*F*_(1/215)_ = 9.469, *p* = 0.002 < 0.05, η^2^ = 0.042], Specifically, users had a more positive PSP of low as opposed to high IA [*M*_*LowIA*_ = 3.70, *SD* = 0.70, *M*_*HighIA*_ = 3.35, *SD* = 0.87], supporting H4b. A nonsignificant interaction effect was obtained [*F*_(1/215)_ = 2.406, *p* = 0.122, η^2^ = 0.011]; thus, H4c was not supported.

The result demonstrated a nonsignificant effect regarding the between-subject effect of AA on PEI [*F*_(1/215)_ = 0.178, *p* = 0.673, η^2^ = 0.01]; thus, H5a was not supported. The result demonstrated a nonsignificant effect regarding the within-subject effect of IA on PEI [*F*_(1/215)_ = 2.611, *p* = 0.108, η^2^ = 0.012]; thus, H5b was not supported. The result revealed a nonsignificant interaction effect [*F*_(1/215)_ = 2.867, *p* = 0.092, η^2^ = 0.013]; thus, H5c was not supported.

H6a proposed that PSP mediates the relationship between PEI and PA. The bootstrapping PROCESS macro in SPSS was utilized to analyze the mediation effects (Hayes, 2022). The analyses were conducted separately for low IA and high IA designs. For low IA designs, the results revealed that the total effect of PEI on PA in the absence of the mediator, PSP, was significant (b = 0.67, t = 8.272, *p* = 0.00 < 0.05, *CI*_95_ = 0.511, 0.830). The indirect effect of PEI on PA via the mediator, PSP, was significant, as the 95% bias-corrected bootstrap confidence interval (based on a 5,000-sample) excluded zero (b = 0.44, t = 4.712, *CI*_95_ = 0.258, 0.625). Furthermore, the direct effect of PEI on PA in the presence of the mediator, PSP, was also found significant (b = 0.23, t = 2.191, *p* = 0.03 < 0.05, *CI*_95_ = 0.023, 0.435), indicating that PSP partially mediated the relationship between PEI and PA. For high IA designs, the results revealed that the total effect of PEI on PA in the absence of the mediator, PSP, was significant (b = 0.77, t = 8.468, *p* = 0.00 < 0.05, *CI*_95_ = 0.590, 0.948). The indirect effect of PEI on PA via the mediator, PSP, was significant, as the 95% bias-corrected bootstrap confidence interval (based on a 5,000-sample) excluded zero (b = 0.58, t = 4.328, *CI*_95_ = 0.321, 0.843). Furthermore, the direct effect of PEI on PA in the presence of the mediator, PSP, was found nonsignificant (b = 0.19, t = 1.379, *p* = 0.169>0.05, *CI*_95_ = –0.081, 0.459), indicating that PSP fully mediated the relationship between PEI and PA. Consequently, based on mediation analyses from both IA design conditions, H6a was partially supported. However, considering PEI was in cross-loading with PSP in both AA conditions according to validity tests via factor analyses, PEI was also not influenced by any robot anthropomorphic variable. Hence, PEI should be removed from the research model.

As PEI was removed from the research model, the direct influence of PSP on PA would need to be tested again. Single linear regression analyses were conducted separately for low IA and high IA designs. Results showed that, for low IA designs [*F*_(1/216)_ = 115.57, *p* < 0.001], PSP (β = 0.816, *p* < 0.001, *R*^2^ = 0.349) positively influences PA, and explained 34.9% of the variance of PA. For high IA designs [*F*_(1/216)_ = 100.69, *p* < 0.001], PSP (β = 0.906, *p* < 0.001, *R*^2^ = 0.318) positively influenced IU and explained 31.8% of the variance of PA. Hence, PSP positively influences PA; the positive model path of PSP to PA was confirmed. As only IA positively influenced PSP, the significant model path IA to PSP to PA was formed. Also, considering the significant main effect of IA on PA, PSP partially mediates the relationship between IA and PA; thus, H6c was partially supported, while H6b and H6d were rejected.

H7 proposed that PA positively influences users' IU. Single linear regression analyses were conducted separately for low IA and high IA designs. Results showed that, for low IA designs [*F*_(1/216)_ = 131.11, *p* < 0.001], PA (β = 0.509, *p* < 0.001, *R*^2^ = 0.378) positively influences IU, and explained 37.5% of the variance of IU. For high IA designs [*F*_(1/216)_ = 355.65, *p* < 0.001], PA (β = 0.567, *p* < 0.001, *R*^2^ = 0.622) positively influenced IU and explained 62.2% of the variance of IU. Hence, H7 was supported.

**Figure 8:**
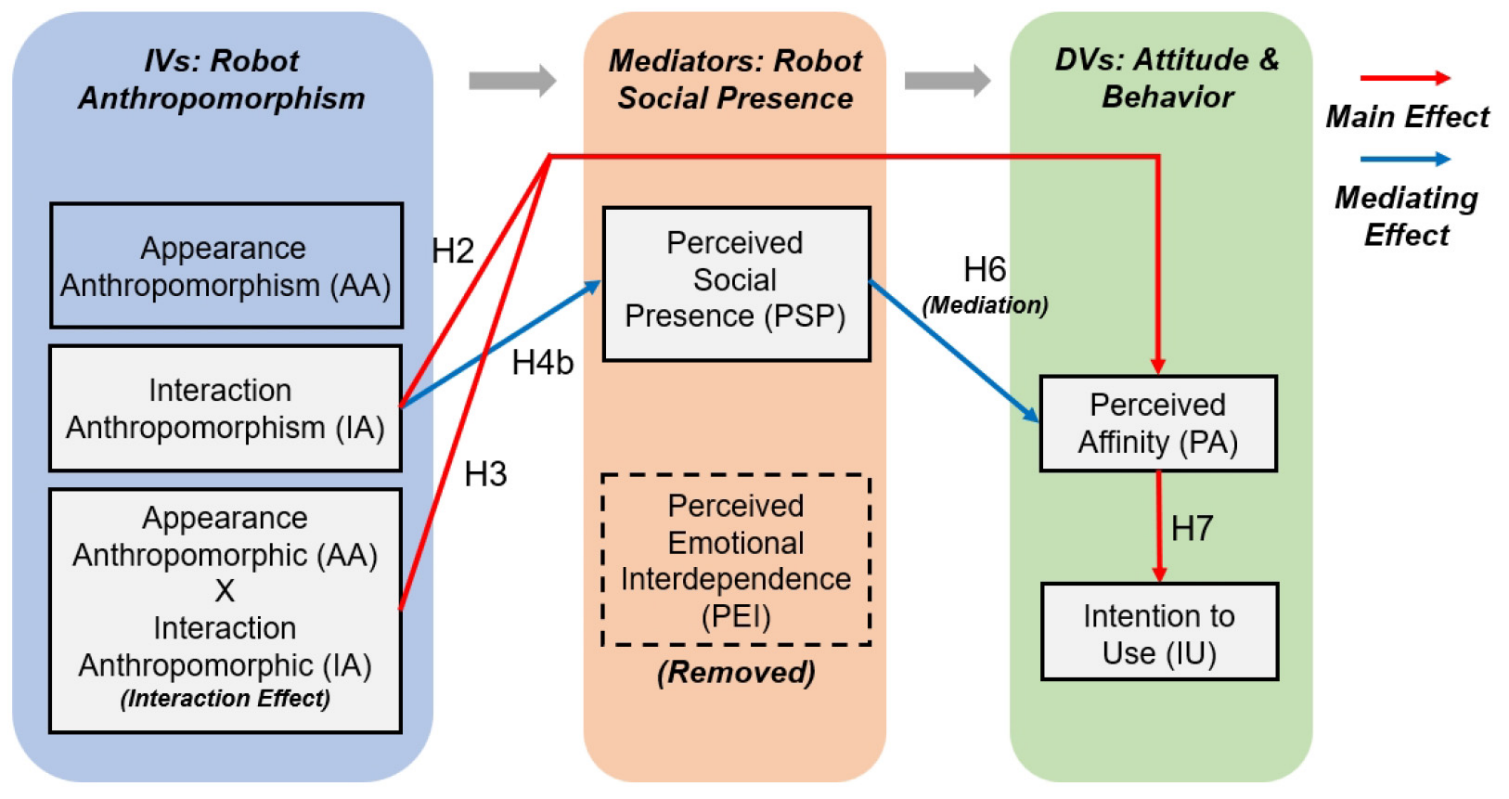
Final research model with supported hypotheses.

The results of all the hypotheses are shown in Table 2, and the final research model with supported hypotheses is shown in Figure 8.

**Table 2:** The results of all the hypotheses.

Hypothesis	Path	Statistics method	Statistics results	Hypothesis supported
H1 H2 H3	AA → PA IA → PA AA × IA → PA	Repeated measures ANCOVA, Covariate: gender	*F* (1/215) = 2.899, *p* = 0.09, η = 0.013. *F*_(1/215)_ = 4.348, *p*=0.038 < 0.05, η = 0.02; *F*_(1/215)_ = 8.546, *p*=0.004 < 0.05, η = 0.038;	Not supported **Supported** **Partially supported**
H4a H4b H4c	AA → PSP IA → PSP AA × IA → PSP	Repeated measures ANCOVA, Covariate: gender	*F*_(1/215)_ = 0.082, *p* = 0.774, η = 0.00. *F*_(1/215)_ = 9.469, *p* = 0.002 < 0.05, η = 0.042; *F*_(1/215)_ = 2.406, *p* = 0.122, η = 0.011.	Not supported **Supported** Not supported
H5a H5b H5c	AA → PEI IA → PEI AA × IA → PEI	Repeated measures ANCOVA, Covariate: gender	*F*_(1/215)_ = 0.178, *p* = 0.673, η = 0.01. *F*_(1/215)_ = 2.611, *p* = 0.108, η = 0.012. *F* (1/215) = 2.867, *p* = 0.092, η = 0.013.	Not supported Not supported Not supported
H6a *(Mediation)* H6b *(Mediation)* H6c *(Mediation)* H6d *(Mediation)* H7	PEI → PSP → PA AA → PSP → PA IA → PSP → PA AA × IA → PSP → PA PA → IU	bootstrapping PROCESS macro in SPSS, Single linear regression N.A. N.A. N.A. Single linear regression	See details in the paragraphs. SP → PA supported. Inferred from H1, H4a and H6a. Inferred from H2, H4b, and H6a. Inferred from H3, H4c and H6a. for low IA designs: *F*_(1/216)_ = 131.11, *p* < 0.001, PA (β = 0.509, *p* < 0.001, *R*^2^ = 0.378);	**Partially supported** Not supported **Partially supported** Not supported **Supported**

Bold values indicate supported or partially supported hypotheses.

## 6 Discussion

Guided by the Robots' Social Presence Modified Model (Chen et al., 2023) and the Theory of Planned Behavior (TPB) (Ajzen, 1991), this study examined the influence of the domestic robot anthropomorphism on users' PA. The findings generally confirmed the feasibility of applying the Robot's Social Presence Modified Model and TPB in explaining the mediating effect of PSP in the relationship between robot anthropomorphism and users' positive attitudes of PA, and further confirmed the positive influence of PA on users' IU. The findings are elaborated in the subsequent paragraphs.

Considering the appearance dimension of robot anthropomorphism, AA was hypothesized to positively influence users' PA. The result indicated a nonsignificant effect of AA on users' PA. This result contradicts prior studies that AA positively affects users' attitudes and PA (Roesler et al., 2024; Goetz et al., 2003; Złotowski et al., 2020). A previous study proposed the task-congruence principle and argued that the design of a robot's appearance should match its functions and work scenarios to evoke users' likability (Li et al., 2010). Hence, the non-significant influence of AA suggests several alternative explanations. A plausible explanation for this discrepancy may stem from the study's stimulus design. This study used static image stimuli may have failed to trigger the dynamic cues necessary for full social attribution in HRI. Users may have perceived the robots' AA not as animate social agents, but as lifeless sculpture. Thus, stimuli with static images may have neutralized the potential positive effects of AA, leading to a nonsignificant influence of AA on users' PA. Alteratively, the high AA stimulus was operationalized as a humanoid torso integrated with a mechanical chassis. This hybrid, semi-anthropomorphic appearance may have lacked sufficient human likeness to elicit perceptions of the robot as a truly human-like entity. Consequently, future research should leverage dynamic stimuli, potentially facilitated by multimedia or advanced AI technologies, to enhance ecological validity. Moreover, the implementation of a high-AA stimulus featuring a complete humanoid form may yield a more significant predictive influence of anthropomorphism on user perception.

With respect to the interaction and facial expression dimension of robot anthropomorphism, IA was hypothesized to positively influence users' PA. The result demonstrated a positive effect of IA on users' PA. This result is in line with prior research showing the positive influence of IA on users' attitudes and PA (Chen and Jia, 2023; Lehmann et al., 2016; Wairagkar et al., 2022). It highlights the importance of robot facial expressions to convey information and emotions, and further enhance HRI. The reason for the significant influence of IA may be that this study demonstrated five facial expressions (i.e., working, greeting, confusion, upsetting, and sleeping) for five modes used in different scenarios, users may feel they were interacting with a real human, thus leading to a positive influence. Results further indicated that, compared to high IA, low IA is more preferable. This finding, to some extent, follows the pattern of the uncanny valley effect (Mori, 1970) and demonstrates that users may perceive high IA facial expressions as strange and unnatural, making them uncomfortable with such domestic robots of mismatched rigid bodies and highly realistic faces in the same space (Kätsyri et al., 2015). Uncanny valley effect may be one of the potential factors to influence IA on users' PA. Hence, RQ1 was partially confirmed.

A significant interaction effect of AA and IA on PA was found. This result supported prior research on the positive interaction effect of AA and IA on users' attitudes toward PA (Wairagkar et al., 2022). The findings were also consistent with the task-congruence principles that a robot's appearance should match its functions and work scenarios to receive users' positive attitudes (Roesler et al., 2024; Goetz et al., 2003). It could be inferred that the facial expressions and interaction conditions underscore the robot's domestic working attributes and working environment, functions of communication, and providing services, thus positively leading to PA. Further, interaction effect plot (see Figure 7) demonstrated that the robot designs generally obey the task-congruence principles (Li et al., 2010; Goetz et al., 2003) and are likely to be influenced by the uncanny valley effect (Mori, 1970; Mori et al., 2012), specifically, even though users generally prefer low IA (compared to high IA), potentially confirming the uncanny valley effect, while the PA of the high AA and high IA condition is higher than the low AA and high IA one, as users may perceive a better match of high human-like robots with the domestic social environment.

Contrary to the hypotheses of mediation effects, besides only H4b was supported, the non-significant influence of AA, IA, or the interaction effect of AA and IA on social presence variables (i.e., PSP and PEI) was examined; the reason can likely be attributed to the same limitation discussed previously that, the study used static images without demonstrating robots' movements. This point also explained the non-significant influence of the interaction effect of AA and IA on PSP and PEI, as realistic facial expressions in a rigid, unmovable body are awkward for users to perceive as an alive human, thus failing to establish social and emotional connections.

PSP, as a key mediator in this study, was predicted to mediate the relationship between PEI and PA. Although the results supported this hypothesis, the PEI construct was subsequently removed from the research model. This decision was based on two key issues: first, PEI exhibited cross-loading with PSP in factor analyses, compromising its discriminant validity; and second, it was not significantly influenced by any of the robot anthropomorphism variables. Previous study suggests that PSP and PEI are theoretically distinct constructs. PEI represents a secondary relational layer, predicated upon sustained affective and dynamic interactions characterized by subtle attitudes and emotional states (Chen et al., 2023). The nonsignificant influence of PEI may be attributed to several reasons. It is probable that the experimental influence was attenuated by the use of static imagery. Given that static image stimuli are inherently limited in their capacity to convey the depth of dynamic HRI and subtle emotional cues, the formation of a secondary interdependent relationship may have been impeded. Consequently, participants may have struggled to differentiate between the constructs of PSP and PEI, potentially leading to statistical cross-loading and the subsequent absent of PEI's unique interaction effect. Hence, it is likely attributable to the methodological constraints, specifically the limitations of the static stimuli, rather than a fundamental absence of the PEI construct within the HRI framework. Alternatively, short-term interaction was likely inadequate for establishing familiarity, trust, and emotional connections for domestic social robots. It is also possible that unmeasured latent variables, such as familiarity, trust, sense of touch, voice interaction, etc., influenced the emotional responses. Consequently, based on the confirmed hypotheses discussed previously, the PSP positively partially mediates the relationship between IA and PA, while not for AA and the interaction effect of AA and IA. Hence, even though PEI was removed from the research model, this study still highlighted the crucial mediating role of social presence and, to some extent, confirmed the feasibility of the robot social presence model to be applied in HRI research. Hence, RQ2 was partially confirmed.

This study hypothesized that users' PA positively influences users' IU of the anthropomorphic social robots. The findings supported the hypothesis and were in line with the TPB model (Ajzen, 1991) and previous studies that attitudes influence their behaviors; users' positive attitudes toward PA of anthropomorphic social robots lead to their IU of the robots in a future family environment.

It is worth noting that, this study used static images as stimuli to study dynamic HRI. While static images share certain attributes with real-time interactions with social robots, they lack the high-fidelity ecological validity required to fully represent dynamic exchanges. Although the hypotheses concerning inherently dynamic constructs (i.e., IA and PSP) were supported in this context, these findings do not inherently guarantee generalizability in dynamic interactions with social robots. Hence, a rigorous interpretation suggests that the supported hypotheses in this study should be viewed as preliminary evidence, indicating effects that are likely or potentially present during interactions with dynamic, embodied social robotics.

## 7 Limitations

This study has several limitations.

Regarding the manipulation quality of robot stimuli, this study was conducted in a multimedia intelligent laboratory environment, using static robot images instead of physically embodied and interactive robots.This method and experimental setup may constrain users from observing dynamic interacting with social robots, but they can only make judgments based on visual perceptions of images and descriptive texts. Even though static imagery can, to a degree, convey the behavioral and affective cues of a social robot (Fan et al., 2023), empirical evidence from psychology and neuroscience suggests that the perception of emotion from static and dynamic stimuli demonstrated distinct neural activation mechanisms (Sato et al., 2004; Weyers et al., 2006). Research further demonstrates that dynamic stimuli, such as body movements and facial expressions, facilitate more accurate and rapid perception (Calvo et al., 2016), enhance the recognition of subtle expressions (Yitzhak et al., 2018), and evoke significantly higher levels of physiological arousal and self-reported emotional valence compared to the static counterparts (Detenber et al., 1998). Consequently, regarding the validated effects of IA and PSP, it is plausible that the magnitude of these influences was underestimated in the current study. Because IA is characterized by dynamic facial expressions and PSP reflects the subjective perception of a human-like interactive agent, both are fundamentally highly interactive, affect-driven constructs. Static image stimuli cannot adequately convey the subtle dynamics of social expressions, the full impact of these variables may only be realized through dynamic interactions. It remains to be determined whether these effects are underestimated concerning statistical significance when interacting with a real dynamic interactive robot. The use of static image stimuli may have also attenuated the effects of AA and PEI, leading to non-significant influences. Future studies require more scientific and rigorous examinations, and may employ more dynamic stimuli and demonstrate innovative robot concepts via multimedia methods, such as audio, animations, AR, and AI technologies, or even real robots, to provide a holistic, multi-sensory and dynamic experience for users. Furthermore, establishing collaborations with industry partners to conduct case studies with physical dynamic interactive robots would yield more robust and generalizable results. Such *in-situ* research would allow for a more rigorous validation of the proposed model and generate greater practical insights.

Regarding the uncanny valley effect, although some of the findings aligns with this theoretical pattern, the limitations of the research design require a cautious interpretation. Because AA and IA were operationalized using binary manipulations (low *vs*. high), attributing these results exclusively to the uncanny valley effect would be premature. To achieve empirical rigor, future studies should implement multi-level manipulations of AA and IA to better predict these non-linear relationships. Furthermore, future studies should investigate additional mediating or moderating variables that may contribute to the non-linear relationships inherent in HRI.

Regarding the research design, this study constrained the robots used in domestic scenarios. Unlike interactions with public service robots, the development of trust and familiarity with domestic social robots is a gradual process; the long-term effects of social presence and emotional interdependence cannot be formed and perceived with a short exposure of the robot stimuli. Thus, it may weaken the results of this study. Future studies may launch longitudinal research and investigate how PSP and PEI are formed in a family environment. The process of making friends and building emotional ties with domestic social robots may play a significant role in influencing users' PSP and PA, and further enhance users' IU.

Regarding the source of the samples, this study used convenient student samples from a university in East China. While student sample ensured a higher degree of technology acceptance, it presents a clear limitation to the external validity of our findings. The assumption that younger populations provide more reliable data due to their openness to new technologies and social robots remains an academic debate and was not empirically tested in this study. Hence, the results may not be generalizable to a broader population. Furthermore, domestic social robots are increasingly designed for a broad population, including older adults and less tech-experienced users. These groups may perceive social presence and anthropomorphic cues differently. For instance, older adults might be more dependent on social robots, prioritize functional reliability over the perceived affinity examined here. Less tech-experienced users may feel technology anxiety and trust barriers, focus on functionalities other than aesthetic appeal. People in Asian countries tend to live together, emphasize family relationships, and may have a higher preference for anthropomorphic robots. Consequently, future studies should prioritize sample diversity and may focus on group differences, such as age, cultural, ethnic and regional differences, to examine the generalizability of our findings. The investigation of the disparities among different demographic groups may result in specific and detailed conclusions and implications, help promote the acceptance of social robots to a broad population and scenarios.

Regarding statistical limitations, it is plausible that the psychometric scales employed lacked the requisite measurement sensitivity to detect subtle psychological shifts within this specific demographic. Alternatively, the current statistical power may have been attenuated by a limited sample size. Future research should implement a rigorous series of pre-tests to validate measurement sensitivity prior to data collection. Additionally, expanding the participant pool to enhance statistical power may ultimately reveal significant effects for AA and PEI.

## 8 Implications

### 8.1 Theoretical implications

This study examined and confirmed the applicability of the Robots' Social Presence Modified Model and TPB to predict users' perceptions and attitudes toward domestic social robots via PSP. A key finding indicated that the main effect of IA positively influenced users' PA. To further enhance HRI research, future studies may explore additional dimensions of IA, such as nuanced facial expressions, body language, voice, or the interaction effects among them. Moreover, subsequent studies may keep highlighting the crucial mediating role of the robot social presence on users' attitudes, explore diverse forms of robot anthropomorphism to improve robots' sociability, and further influence users' PA.

It is noteworthy that the hypotheses related to AA and the mediating role of PEI were not supported. Previous studies (Li et al., 2010; Goetz et al., 2003) indicated the task-congruence principle that AA may influence users' attitudes when AA matches its working scenarios. Thus, future studies may examine feasible AA designs to match the family environment or explore different robot service scenarios to find matching AA designs to positively influence users' PA and IU. An alternative reason may be that AA stimuli were static images without showing robots' movements. Future research may develop dynamic robot stimuli or physical robot samples that enable users' multi-sensory perceptions of robot AA, such as the demonstrations of human-like movements and gestures, which may be necessary to elicit a positive effect from AA.

The PEI was in cross-loading with PSP and was not significantly influenced by any of the robot anthropomorphic factors; thus, it was removed from the research model. Future studies may focus on refining the instruments of PSP and PEI to ensure these concepts are clearly delineated and manipulating higher-quality stimuli to better represent robot anthropomorphism. Future studies may also explore potential latent variables influencing emotions, such as familiarity, trust, sense of touch, voice interaction, etc., to reveal the mechanism of emotional connections and find the significant mediating influence of PEI in HRI.

It is plausible that the high IA stimuli manipulated in this study inadvertently exceeded the boundary and fell into the uncanny valley, leading to a lower preference for high IA. Consequently, a crucial avenue for future research is to explore the boundary of the uncanny valley effect for specific domestic scenarios under contemporary technologies. Such an investigation is essential for guiding the design of highly anthropomorphic robots that can elicit positive user evaluations while carefully avoiding the negative influence associated with the uncanny valley effect.

The demonstrated viability of employing the Robots' Social Presence Modified Model for designing anthropomorphic robots offers significant theoretical implications for the field. This finding calls for continued research investigation focused on the model, including rigorous testing of unsupported hypotheses and variables. To achieve a holistic view of the pivotal mediating function of social presence in robot anthropomorphism, future work should also endeavor to identify and analyze additional mediating factors and dimensions. Pursuing this research agenda will serve to substantively advance and enrich the study of HRI.

### 8.2 Practical implications

The significant main effect of IA on users' PA and use intentions provides practical implications for robot scientists and manufacturers. Given the current nascent stage of social robot adoption, a strategic focus on low IA is advisable. Designs that employ minimalist or schematic features, such as simple lines and circles to represent facial expressions, can be highly effective. This approach serves a dual purpose: it avoids the risk of inducing an uncanny valley effect and ensures that a robot's anthropomorphic cues are readily interpretable, thereby fostering greater public acceptance and paving the way for broader application.

In light of the significant mediating effect of PSP, the finding provides a clear direction for HRI development: making domestic social robots look like and interact like authentic human partners is a fundamental task for robot scientists and manufacturers. In the future, designing the functions of social robots that enhance human multi-sensory interactions, such as voice communications, body language, or sense of touch, could enable users to feel the social presence of the domestic social robots as real humans, and this will be critical for promoting the social robots into daily services. Subramanian et al. (2024) study provided an alternative solution to improve HRI via augmented reality (AR) technology. AR technology could provide visual enhancement to enable users to feel social collaboration and safety during HRI, as if working with a real person. By leveraging technologies like AR to bolster PSP, designers can positively influence users' PA and willingness to use social robotic systems.

The significant interaction effect of AA and IA on PA holds crucial implications for future HRI design. As shown in the interaction plots (see Figure 7), in high IA conditions, the users' PA increased from low AA to high AA. This suggests that, in the future, as users gain familiarity with social robots through widespread applications in daily lives, the aversion related to the uncanny valley effect may be overcome. Consequently, anthropomorphic robots, high in both AA and IA, have the potential to eventually achieve superior user evaluations. Therefore, continuing to explore new frontier technologies and designing high anthropomorphic robots with better functionalities may potentially cater to the domestic social interaction demands of users, enhance HRI, and benefit our society in the long term.

The intelligent design process was proven to facilitate the design of robot anthropomorphism. In contrast to the traditional product design workflows—from initial ideation to 2D sketches and 3D modeling, which are often time-intensive processes requiring days or weeks, intelligent design process leverages emerging technologies such as AI and latest Apps. It allows for the rapid generation of numerous high-quality images, inspiring design ideas and providing high-quality materials for robot anthropomorphism manipulations. It is recommended that future robot design continues to adopt the intelligent design process, further integrating and exploring new technologies to elevate overall design quality and efficiency.

## Data Availability

The raw data supporting the conclusions of this article will be made available by the authors, without undue reservation.

## Appendix

**Table A1:** Measurements.

Variable	Measurements	Reported Reliability
Appearance	3 items, 7-point Likert scale, adapted from (David et al. 2022)	Cronbach's α = 0.89
	For robot appearance anthropomorphism	
	(1) The robot looks like a human.	
	(2) The robot looks like a person.	
	(3) The robot is human-like.	
	For robot interaction anthropomorphism	
	(1) The robot's facial expressions look like those of a human.	
	(2) The robot's facial expressions look like those of a person.	
	(3) The robot's facial expressions are human-like.	
Perceived affinity	8 items, 7-point Likert scale, adapted from (Belanche et al. 2021)	Composite reliability (CR) = 0.913 AVE = 0.687
	(1) I think that the robot is likable.	
	(2) I think that the robot is attractive.	
	(3) I think that the robot is familiar.	
	(4) I think that the robot is natural.	
	(5) I think that the robot is intelligent.	
	(6) I think that the robot is warm.	
	(7) I think that the robot is nice.	
	(8) I think that the robot is good.	
Intention to use	4 items, 5-point Likert scale, adapted from (Debasa et al. 2023)	Cronbach's α = 0.818, composite reliability (CR) = 0.882, AVE = 0.654
	(1) I will recommend the use of the service robot to my family and friends.	
	(2) I hope to improve my quality of life by using the service robot.	
	(3) I intend to use the service robot as often as necessary.	
	(4) I hope to use the service robot in the future.	
Perceived social presence	5 items, 5-point Likert scale, adapted from (Chen et al. 2023)	Cronbach's α = 0.851, Composite reliability (CR) = 0.850, AVE = 0.535
	(1) During interaction with the social robot, I noticed it.	
	(2) The presence of social robots is obvious to me.	
	(3) I think social robots receive my attention in interactions.	
	(4) I think that when interacting with robots, we coexist in the space of the present moment.	
	(5) I keep an eye on the social robots as I interact with them.	
Perceived emotional interdependence	3 items, 5-point Likert scale, adapted from (Chen et al. 2023)	Cronbach's α = 0.749, Composite reliability (CR) = 0.821, AVE = 0.610
	(1) The attitude of the robot during the interaction influenced my feelings.	
	(2) I felt the atmosphere between us during the interaction. (3) I think the social robots in our interactions affected how I felt.	
